# NLRP3 Inhibitor KBD3536 Attenuates Acute Inflammation, Radiation-Induced Skin Injury, and Early Metabolic Dysfunction in Preclinical Models

**DOI:** 10.3390/ph19071083

**Published:** 2026-07-14

**Authors:** Xinying Qian, Fei Ye, Zhiyong Li, Hongzhu Chu, Zeng Xu, Wenyuan Peng, Xueya Liang, Hongchuan Zhao, Yan Tang, Pan Zhong, Yonggang Wei, Yinglan Zhao

**Affiliations:** 1 Department of Biotherapy, Cancer Center and State Key Laboratory of Biotherapy, West China Hospital, Sichuan University, Chengdu 610041, China; qianxinying@stu.scu.edu.cn; 2 Kangbaida (Sichuan) Biotechnology Co., Ltd., Chengdu 611137, China; yefei@baiyu.cn (F.Y.); lizhiyong@baiyu.cn (Z.L.); chuhz@baiyu.cn (H.C.); xuzeng@baiyu.cn (Z.X.); pengwenyuan@baiyu.cn (W.P.); liangxueya@baiyu.cn (X.L.); zhaohc@baiyu.cn (H.Z.); tangyan@baiyu.cn (Y.T.); asadhu@163.com (P.Z.)

**Keywords:** NLRP3 inhibitor, obesity, gouty arthritis, radiation dermatitis, KBD3536

## Abstract

**Background**: Pharmacological blockade of the NOD-like receptor family pyrin domain-containing 3 (NLRP3) inflammasome has emerged as an attractive pharmacological strategy for a broad range of inflammatory and metabolic disorders. However, translating preclinical efficacy into clinical success remains a major bottleneck. We previously reported the discovery of a novel, potent NLRP3 inhibitor, KBD3536, but its in vivo efficacy across different pathological conditions remains uncharacterized. Here, we systematically evaluated the in vivo efficacy of KBD3536 across diverse preclinical models of NLRP3-related pathologies. **Methods**: KBD3536 was evaluated in established rodent models of monosodium urate (MSU)-induced acute inflammation (mouse air pouch and rat gouty arthritis models), radiation-induced dermatitis (RID), and high-fat diet (HFD)-induced obesity. **Results**: In the MSU models, KBD3536 markedly suppressed local interleukin-1β and interleukin-6 secretion in air pouch exudates and dose-dependently alleviated acute arthritis symptoms, including joint swelling and joint pain. In the RID model, KBD3536 significantly attenuated radiation-induced skin injury and ameliorated radiation-induced systemic weight loss. Under HFD challenge, early intervention with KBD3536 mitigated HFD-induced adiposity, early hepatic steatosis and its associated inflammatory responses, and preserved physical performance. Mechanistically, KBD3536 partially restored AMP-activated protein kinase α1 (AMPKα1) mRNA expression in adipose tissue and restored hepatic *Cyp3a11* transcriptional activity. **Conclusions**: NLRP3 inhibitor KBD3536 exhibited broad-spectrum anti-inflammatory efficacy across multiple preclinical models, supporting its potential as a promising candidate for diverse NLRP3-related disorders.

## 1. Introduction

The innate immune system serves as the first line of defense against cellular stress and exogenous insults. A key component of this defense network is the NOD-like receptor family pyrin domain-containing 3 (NLRP3) inflammasome, an intracellular multiprotein complex involved in homeostatic surveillance [1,2,3]. The NLRP3 inflammasome comprises the NLRP3 sensor, the adaptor protein ASC (apoptosis-associated speck-like protein containing a CARD), and the effector protease caspase-1 [4]. Upon activation by diverse danger signals, this complex mediates the proteolytic maturation and secretion of the pro-inflammatory cytokines interleukin-1β (IL-1β) and interleukin-18 (IL-18), while also triggering pyroptosis, an inflammatory form of programmed cell death [5,6]. Historically, gain-of-function NLRP3 mutations were associated with cryopyrin-associated periodic syndromes (CAPS), a group of rare hereditary autoinflammatory disorders [7,8]. It is now clear, however, that aberrant activation of the wild-type NLRP3 inflammasome contributes to a broad spectrum of inflammatory pathologies, ranging from acute gouty flares to pathological tissue remodeling and chronic metabolic disorders [9,10,11].

The NLRP3 inflammasome can be activated by a structurally diverse array of stimuli, broadly categorized into acute crystalline danger-associated molecular patterns (DAMPs), physical or environmental stress, and chronic metabolic overload [12,13,14]. Aberrant NLRP3 activation is therefore implicated in a range of pathological conditions with substantial unmet clinical needs [11]. In acute inflammatory settings, crystalline DAMPs such as monosodium urate (MSU) trigger gouty arthritis, for which current therapies often fail to provide rapid and adequate pain control during acute flares [15,16]. Physical stressors such as ionizing radiation can trigger severe radiation-induced dermatitis (RID), a painful radiotherapy-induced complication for which no targeted therapy currently exists to halt local tissue injury and subsequent fibrogenic remodeling [17]. Under chronic metabolic overload, prolonged overnutrition, often driven by obesogenic diets, promotes low-grade meta-inflammation, leading to obesity and a spectrum of metabolic complications, including cardiovascular and hepatic dysfunction. However, current therapeutic strategies predominantly target late-stage metabolic symptoms rather than mitigating the early, NLRP3-driven meta-inflammation that precedes severe metabolic dysfunction [18,19,20,21,22,23,24].

Accordingly, given the broad pathological relevance of aberrant NLRP3 activation, pharmacological blockade of the NLRP3 inflammasome has emerged as an attractive therapeutic strategy. Over the past decade, several small-molecule NLRP3 inhibitors, including selnoflast (Roche) [25], NT-0796 (NodThera) [26,27], AZD4144 (AstraZeneca) [28], VTX-3232/VTX2735 (Ventyx Biosciences/Eli Lilly) [29,30,31], and ZYIL-1 (Zydus Cadila) [32,33,34] have advanced into Phase II clinical trials for a range of inflammatory indications, including atherosclerosis, obesity, sepsis-associated acute kidney injury, Parkinson’s disease, amyotrophic lateral sclerosis, and ulcerative colitis. Despite these advances, no NLRP3 inhibitor has yet been approved, largely owing to the challenge of translating promising preclinical efficacy into sustained and safe clinical benefit. There thus remains a need to explore inhibitors with distinct chemical structures and to rigorously evaluate their pharmacodynamics across highly relevant preclinical disease models. Such in vivo profiling is important for building a more robust foundation for the clinical advancement of NLRP3-targeted therapeutics.

In our recent study, we identified KBD3536 (previously designated compound 32) as a novel and potent small-molecule NLRP3 inhibitor [35]. KBD3536 demonstrated robust in vitro inhibitory activity, with an IC_50_ value of 27.7 nM in the THP-1 pyroptosis assay and an IC_50_ of 19.5 nM for inhibition of IL-1β secretion in LPS- and ATP-stimulated peripheral blood mononuclear cells, alongside favorable drug-like properties [35]. Its in vivo efficacy, however, has not been systematically assessed. Here, we systematically evaluated the in vivo potential of KBD3536 in three distinct NLRP3-relavant disease models: MSU-induced acute inflammation, RID, and high-fat diet (HFD)-induced obesity.

## 2. Results

### 2.1. KBD3536 Suppresses MSU-Induced Local and Articular Inflammation In Vivo

**Figure 1:**
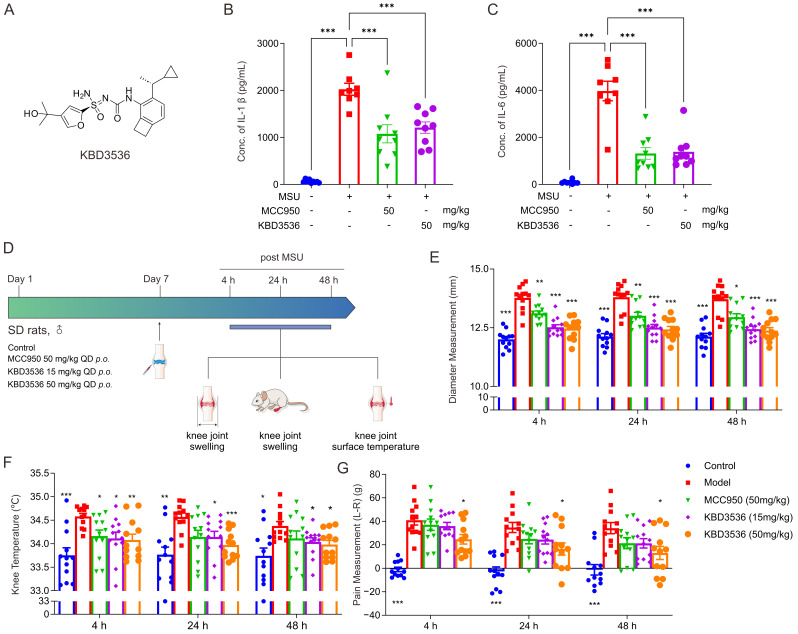
Chemical structure of KBD3536 and in vivo pharmacodynamic validation in monosodium urate (MSU)-induced acute inflammation models. (**A**) Chemical structure of KBD3536. (**B**,**C**) In vivo pharmacodynamic validation in the MSU-induced mouse air pouch model. C57BL/6J mice were assigned to a Control group (no MSU challenge) or treated with vehicle (Model), MCC950 (50 mg/kg), or KBD3536 (50 mg/kg) twice daily (BID) for 3 days before MSU crystal challenge. Concentrations of the pro-inflammatory cytokines (**B**) IL-1β and (**C**) IL-6 in pouch exudates (collected 5 h post-MSU injection) were quantified by ELISA (*n* = 9 per group, except for the Model group in panel B, and both the Control and Model groups in panel C, where *n* = 8 due to technical failure during sample processing). (**D**–**G**) The efficacy of KBD3536 in an MSU crystal-induced rat model of acute gouty arthritis. (**D**) Schematic overview of the experimental design and timeline. Rats received oral treatment with vehicle, MCC950 (50 mg/kg), or KBD3536 (15 or 50 mg/kg) for 9 days, with intra-articular MSU crystal injection on Day 7 to induce acute arthritis. Acute inflammatory and pain responses were evaluated at 4 h, 24 h, and 48 h post-MSU injection, including: (**E**) knee joint swelling assessed by joint diameter, (**F**) local inflammation by knee joint surface temperature, and (**G**) gout-associated joint pain by dynamic weight-bearing incapacitance testing (*n* = 12 per group). Data are presented as mean ± SEM. Individual data points are shown as overlaid symbols. Statistical significance was analyzed using an ordinary one-way ANOVA for (**B**,**C**) and a repeated measures (RM) two-way ANOVA with the Geisser-Greenhouse correction for (**E**–**G**). Both were followed by Dunnett’s multiple comparisons test. * *p* < 0.05, ** *p* < 0.01, *** *p* < 0.001 vs. the MSU model group.

To validate whether KBD3536 effectively inhibits NLRP3 inflammasome activity in vivo (Figure 1A), we employed the classical MSU crystal-induced mouse air pouch model [36]. In the model group, MSU challenge significantly increased IL-1β and interleukin-6 (IL-6) levels in pouch exudates. Oral administration of KBD3536 (50 mg/kg, twice daily [BID]) significantly reduced both IL-1β (Figure 1B) and IL-6 (Figure 1C) concentrations, with inhibitory effects comparable to those of the benchmark NLRP3 inhibitor MCC950.

Following confirmation of local cytokine suppression, we evaluated the preventive and early-intervention efficacy of KBD3536 in an MSU crystal-induced rat model of acute gouty arthritis (Figure 1D). KBD3536 administration at both tested doses (15 and 50 mg/kg) significantly reduced joint swelling across all monitored time points (Figure 1E). KBD3536 also attenuated local temperature increases in a time- and dose-dependent manner (Figure 1F). Both the high dose (50 mg/kg) and the low dose (15 mg/kg) significantly reduced knee temperature across all monitored time points (4 h, 24 h and 48 h). By contrast, the reference drug MCC950 produced a significant effect only during the acute phase at 4 h. These findings indicate that KBD3536 alleviates acute joint inflammation.

To further evaluate functional impairment, we performed dynamic weight-bearing incapacitance testing to assess gout-associated joint pain. Intra-articular MSU injection induced marked weight-bearing asymmetry in the model group, reflected by a weight-bearing difference between the affected and contralateral hind paws. KBD3536 administration restored weight-bearing capacity in the affected limbs in a dose-dependent manner. The high-dose KBD3536 group (50 mg/kg) showed a significantly greater analgesic effect than MCC950 at the same dose, reducing pain-related deficits throughout the monitoring period (Figure 1G).

Throughout the experimental period, animals treated with KBD3536 maintained stable body weights comparable to the control group, indicating no adverse effect on body weight under these conditions (Figure S1). These findings suggest that KBD3536 attenuates MSU-induced inflammatory responses and alleviates acute gout flares in vivo.

### 2.2. KBD3536 Alleviates Radiation-Induced Dermatitis (RID) and Ameliorates Systemic Inflammatory Responses in Mice

**Figure 2:**
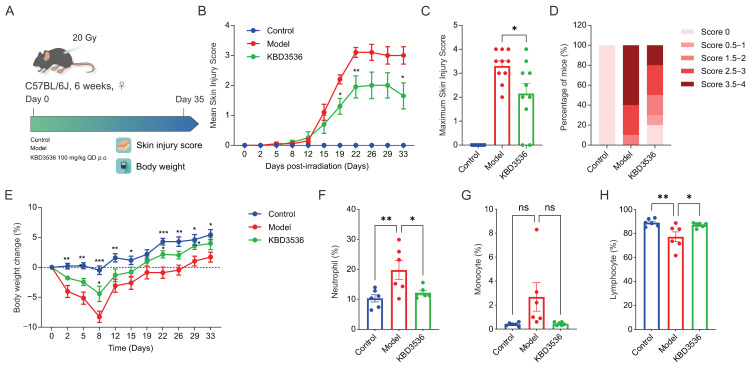
KBD3536 alleviates radiation-induced dermatitis (RID) and associated systemic inflammatory responses in mice. (**A**) Schematic overview of the experimental design. Six-week-old female C57BL/6J mice received a single local X-ray irradiation dose (20 Gy) on Day 0, followed by daily oral treatment with vehicle or KBD3536 for 35 days. (**B**) Clinical dermatitis scores were assessed twice weekly throughout the intervention period. (**C**) Maximum dermatitis score recorded during the experimental period. (**D**) Percentage distribution of dermatitis severity grades. (**E**) Changes in body weight (%) monitored throughout the experiment. Percentages of (**F**) Neutrophils (Neu%), (**G**) Monocytes (Mon%), and (**H**) Lymphocytes (Lym%) in peripheral blood were evaluated using a hematology analyzer at the experimental endpoint. Data are presented as mean ± SEM (*n* = 10 per group for (**B**–**E**); *n* = 6 per group for (**F**–**H**)). Individual data points are shown as overlaid symbols. Statistical significance was analyzed using the Mann–Whitney U test for (**B**,**C**); a RM two-way ANOVA with the Geisser-Greenhouse correction for (**E**); and an ordinary one-way ANOVA for (**F**–**H**). The ANOVA analyses were followed by Dunnett’s multiple comparisons test. * *p* < 0.05, ** *p* < 0.01, *** *p* < 0.001 vs. model group; ns, not significant.

Having demonstrated the anti-inflammatory efficacy of KBD3536 in MSU-induced inflammation, we next investigated its early intervention effects in physical stress-driven tissue injury. Given that ionizing radiation is known to act as a potent physical stressor that activates the NLRP3 inflammasome [37], we established a RID mouse model using a single 20 Gy dose of X-ray irradiation (Figure 2A).

Following irradiation, KBD3536 administration attenuated the progressive skin damage observed in the model group. A downward trend in skin injury scores was apparent from day 19 post-irradiation onward, with statistically significant reductions observed specifically on days 19, 22, and 33 (Figure 2B). Consistent with these findings, the Area Under the Curve (AUC) analysis for the dermatitis scores demonstrated a significant decrease in the KBD3536-treated group compared to the model group (Figure S2). During the monitoring period, KBD3536-treated mice also exhibited a significantly reduced maximal skin injury score compared to the model group (Figure 2C). Grading distribution analysis further indicated that, whereas most model mice developed severe dermatitis (scores ≥ 3.5), KBD3536 treatment shifted the population distribution toward milder clinical presentations (Figure 2D).

To further assess the systemic effects of KBD3536 following irradiation, we evaluated body weight changes and endpoint hematological profiles. KBD3536 administration mitigated radiation-induced body weight loss throughout the experimental period (Figure 2E). Consistently, KBD3536 significantly reduced the elevated proportion of circulating neutrophils (Figure 2F). A similar normalizing trend was observed for monocytes, although this did not reach statistical significance (Figure 2G). Meanwhile, lymphocyte proportions were restored to near-normal levels (Figure 2H). Together, these findings suggest that KBD3536 mitigates both local radiation-induced skin injury and associated systemic inflammatory responses.

### 2.3. KBD3536 Mitigates Radiation-Induced Skin Pathology by Suppressing the Local NLRP3/IL-1β Axis

**Figure 3:**
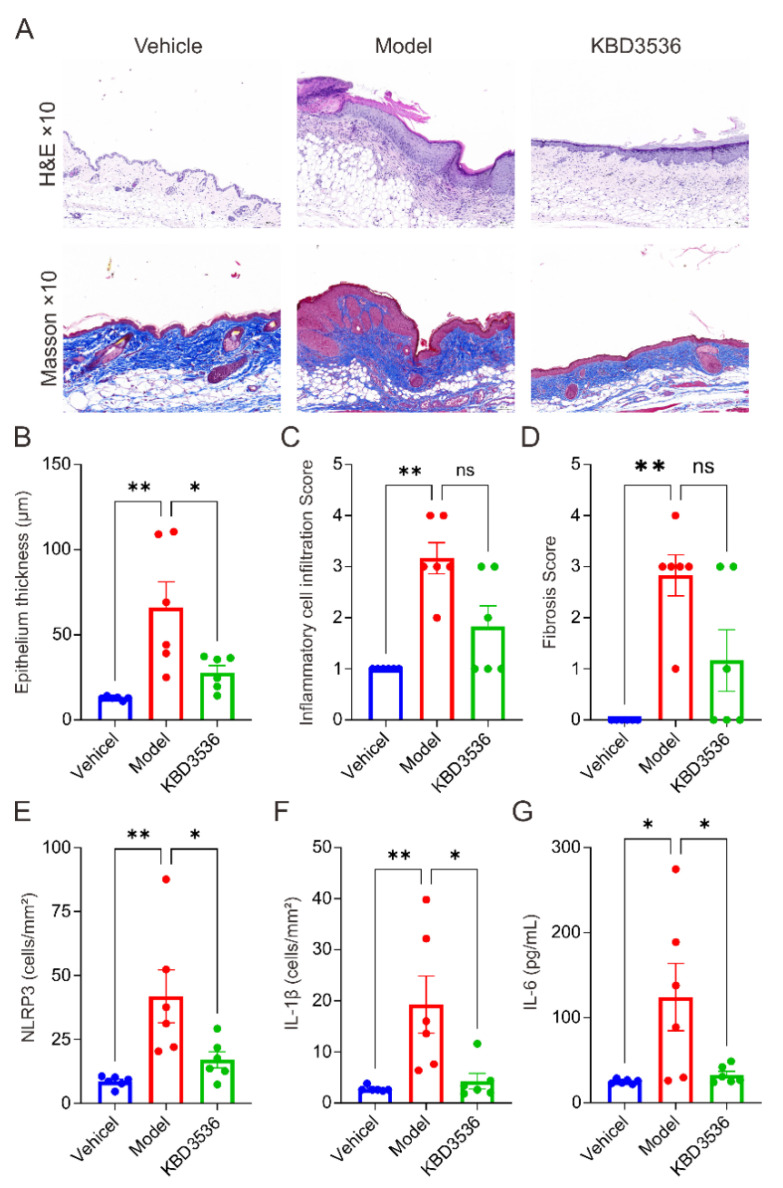
KBD3536 mitigates radiation-induced dermal pathology and suppressing the local NLRP3/IL-1β axis. (**A**) Representative H&E and Masson’s trichrome staining images of irradiated skin tissues (scale bar = 100 μm). In Masson's trichrome staining, collagen fibers are stained blue, while the cytoplasm and muscle fibers are stained red. Histological quantification of (**B**) epidermal thickness, (**C**) inflammatory cell infiltration (score 0–5), and (**D**) dermal fibrosis assessed using the modified Ashcroft score. Quantitative analysis of (**E**) NLRP3- and (**F**) IL-1β-positive cell density (cells/mm^2^) in skin tissues by immunohistochemistry (IHC). (**G**) IL-6 protein levels in skin tissue homogenates quantified by ELISA. Data are presented as mean ± SEM (*n* = 6 per group). Individual data points are shown as overlaid symbols. Statistical significance was analyzed using an ordinary one-way ANOVA followed by Dunnett’s multiple comparisons test for (**B**,**E**–**G**), and the Kruskal–Wallis test followed by Dunn’s multiple comparisons test for (**C**,**D**). * *p* < 0.05, ** *p* < 0.01 vs. model group; ns, not significant.

To characterize the pathological features of this RID mouse model, we performed histological analyses of irradiated skin tissues. Representative H&E and Masson’s trichrome staining images indicated that KBD3536 mitigated radiation-induced skin pathology (Figure 3A). Quantitative analysis of H&E-stained sections further showed that KBD3536 significantly reduced epidermal hyperplasia (Figure 3B). Furthermore, despite not reaching statistical significance—possibly due to the small sample size per group—KBD3536 showed a tendency toward reducing dermal inflammatory cell infiltration (Figure 3C) and collagen deposition (Figure 3D), suggesting a potential to attenuate dermal fibrotic progression in irradiated mice.

To explore the molecular basis underlying these structural effects, we next examined the local NLRP3 signaling pathway. Immunohistochemistry (IHC) analysis revealed that KBD3536 suppressed radiation-induced local overexpression of both NLRP3 (Figure 3E) and its downstream effector IL-1β (Figure 3F), with representative tissue images shown in Figure S3. This localized NLRP3-related inhibition was further corroborated by ELISA results, which demonstrated significantly reduced levels of the downstream pro-inflammatory cytokine IL-6 in skin tissues following KBD3536 treatment (Figure 3G). Together, these findings suggest that KBD3536 attenuates structural alterations and local inflammation in irradiated skin tissues, at least in part, through suppression of the local NLRP3/IL-1β inflammatory axis.

### 2.4. KBD3536 Effectively Attenuates HFD-Induced Weight Gain

**Figure 4:**
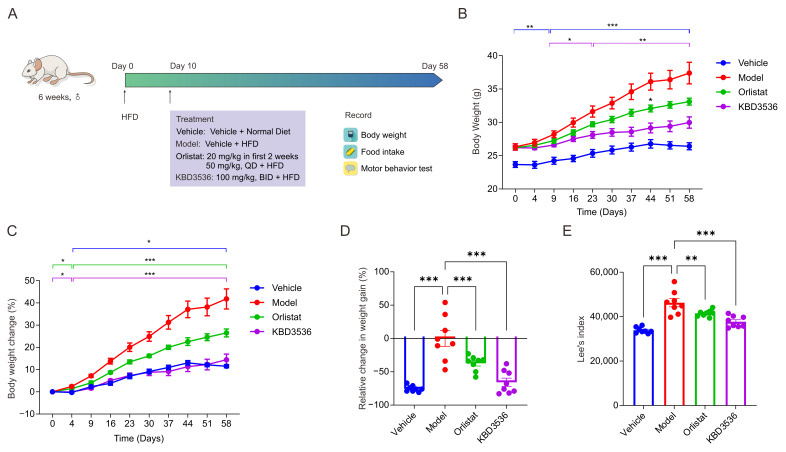
KBD3536 effectively attenuates high-fat diet (HFD)-induced Weight Gain in mice. (**A**) Schematic overview of the experimental design and treatment timeline. Six-week-old male C57BL/6J mice were fed either a standard chow diet or a 60% kcal HFD. Following the emergence of a statistically significant difference in body weight, HFD-fed mice were randomized to receive vehicle (Model group), orlistat (20 mg/kg QD for the first 2 weeks, followed by 50 mg/kg QD), or KBD3536 (100 mg/kg BID) for 58 days (*n* = 8 per group). (**B**) Absolute body weight and (**C**) body weight change rate (%) were monitored throughout the 58-day treatment period. (**D**) Relative change in weight gain (%) and (**E**) Lee’s index were assessed at the experimental endpoint following overnight fasting. Data are presented as mean ± SEM. Individual data points are shown as overlaid symbols. Statistical significance was analyzed using a RM two-way ANOVA with the Geisser-Greenhouse correction for (**B**,**C**), and an ordinary one-way ANOVA for (**D**,**E**). Both analyses were followed by Dunnett’s multiple comparisons test. * *p* < 0.05, ** *p* < 0.01, *** *p* < 0.001 vs. model group.

Given the efficacy of KBD3536 in acute inflammatory models, we next investigated its early-intervention potential in diet-induced obesity. Emerging evidence suggests that aberrant NLRP3 inflammasome activation contributes to obesity-associated chronic inflammation, making it a promising pharmacological target for obesity-related metabolic dysfunction [23,38,39,40]. To evaluate the efficacy of early NLRP3 inhibition in this context, we employed an HFD-induced mouse model. HFD-fed mice were randomized and received early-interventions immediately upon a significant weight divergence from the normal chow-fed vehicle group (Figure 4A). The clinically approved anti-obesity agent orlistat, a well-established lipase inhibitor [41], was included as a positive control.

During the 58-day intervention period, the model group exhibited a progressive increase in both absolute body weight (Figure 4B) and body weight change rate (Figure 4C). Early intervention with KBD3536 (100 mg/kg, BID) significantly attenuated HFD-induced weight gain from day 9 onward and showed a more favorable weight control trend than orlistat (Figure 4B,C). At the study endpoint, KBD3536 treatment significantly reduced overall weight gain, approaching levels observed in the vehicle group (Figure 4D). Consistent with these findings, KBD3536 also significantly reduced Lee’s index, a classical morphometric indicator of obesity (Figure 4E). Importantly, KBD3536 treatment did not significantly alter average daily food intake relative to the model group throughout the experimental period (Figure S4).

In parallel, a 14-day short-term tolerability study showed that KBD3536 at this dose did not alter body weight trajectories or food intake in normal lean mice (Figure S5), with no observable signs of behavioral or physical distress. Collectively, these findings suggest that KBD3536 attenuates HFD-induced weight gain without detrimentally affecting normal physiological growth.

### 2.5. KBD3536 Restricts Adipose Tissue Expansion

**Figure 5:**
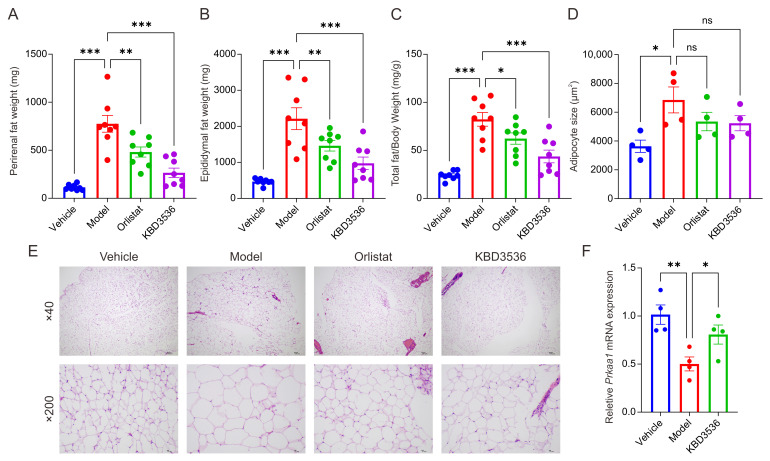
KBD3536 restricts adipose tissue expansion and restores the AMPKα1 mRNA expression in HFD-fed mice. (**A**,**B**) Absolute weights of (**A**) perirenal white adipose tissue (pWAT) and (**B**) epididymal white adipose tissue (eWAT) collected at the experimental endpoint. (**C**) Total fat-to-body weight ratio, calculated as the combined weight of eWAT and pWAT relative to total body weight. (**D**) Quantitative analysis of average adipocyte surface area in eWAT sections, calculated from at least three random fields per mouse. (**E**) Representative H&E staining images of eWAT (scale bars: 100 μm for ×40, and 10 μm for ×200). (**F**) Relative *Prkaa1* mRNA expression (encoding AMPKα1) in eWAT, determined by RT-qPCR and normalized to *Actb*. Data are presented as mean ± SEM (*n* = 8 per group for (**A**–**C**); *n* = 4 per group for (**D**,**F**)). Individual data points are shown as overlaid symbols. Statistical significance was analyzed by ordinary one-way ANOVA followed by Dunnett’s multiple comparisons test. * *p* < 0.05, ** *p* < 0.01, *** *p* < 0.001 vs. model group; ns, not significant.

Excessive weight gain in obesity is largely driven by the accumulation and expansion of white adipose tissue (WAT) [42,43]. At the experimental endpoint, KBD3536 treatment significantly attenuated the expansion of major fat depots compared with the HFD-fed model group. Specifically, KBD3536 reduced the absolute weights of both perirenal (pWAT) and epididymal white adipose tissue (eWAT) (Figure 5A,B), resulting in a lower overall fat-to-body weight ratio (Figure 5C). KBD3536 also showed a favorable trend compared with orlistat.

To further assess these effects at the cellular level, we performed histological analysis of eWAT. Although statistical significance was not achieved—likely due to the limited sample size—KBD3536 exhibited a notable trend toward restricting the HFD-induced increase in adipocyte size (Figure 5D), as corroborated by representative H&E staining (Figure 5E).

To investigate the metabolic pathways potentially underlying these anti-adipogenic effects, we assessed the expression of AMP-activated protein kinase α1 (AMPKα1), a key metabolic sensor [44]. Quantitative real-time PCR (RT-qPCR) analysis revealed that HFD significantly suppressed *Prkaa1* (encoding AMPKα1) mRNA expression in adipose tissue, whereas KBD3536 treatment partially restored its expression (Figure 5F). Together, these findings suggest that KBD3536 suppresses excessive WAT expansion, accompanied by the partial restoration of HFD-suppressed AMPKα1 mRNA expression.

### 2.6. KBD3536 Mitigates HFD-Induced Declines in Physical Performance

Obesity and associated chronic low-grade inflammation are frequently accompanied by the impairment of musculoskeletal quality and reduced exercise performance [45,46]. To evaluate the effects of KBD3536 on musculoskeletal function, we conducted a series of in vivo assessments, including grip strength, rotarod, and treadmill exhaustion testing.

**Figure 6:**
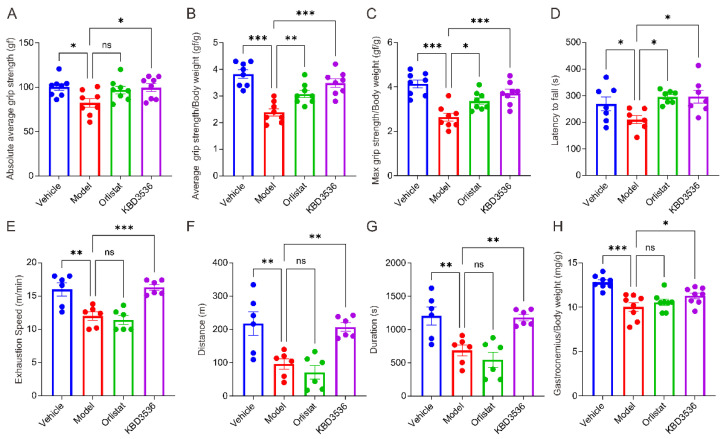
KBD3536 mitigates HFD-induced declines in physical performance and relative muscle mass. (**A**–**C**) Forelimb grip strength was assessed after 6 weeks of treatment, including (**A**) mean absolute grip strength, (**B**) mean grip strength-to-body weight ratio, and (**C**) maximal grip strength-to-body weight ratio. (**D**) Latency to fall in the rotarod test assessed at week 7. (**E**–**G**) Treadmill exhaustion performance at week 8, including (**E**) total running distance, (**F**) total running time, and (**G**) exhaustion speed. (**H**) Gastrocnemius-to-body weight ratio measured at the experimental endpoint. Data are presented as mean ± SEM (*n* = 6 per group for (**A**–**G**); *n* = 8 per group for (**H**)). Individual data points are shown as overlaid symbols. Statistical significance was analyzed by ordinary one-way ANOVA followed by Dunnett’s multiple comparisons test. * *p* < 0.05, ** *p* < 0.01, *** *p* < 0.001 vs. model group; ns, not significant.

In grip strength assessments, KBD3536 significantly improved HFD-induced reductions in mean absolute grip strength (Figure 6A), mean grip strength-to-body weight ratio (Figure 6B), and maximal grip strength-to-body weight ratio (Figure 6C), demonstrating a favorable trend compared with orlistat. Similarly, in the rotarod test assessing motor coordination, both KBD3536 and orlistat prolonged the reduced latency to fall induced by chronic HFD feeding (Figure 6D).

Differences between treatments became more apparent during the treadmill exhaustion test. Whereas orlistat showed limited effects on HFD-induced declines in total running distance, duration, and exhaustion speed, KBD3536 improved all three endurance-related measures (Figure 6E–G). These findings suggest a greater preservation of exercise endurance and physical capacity with KBD3536 treatment.

Consistent with these functional findings, KBD3536 significantly attenuated the HFD-induced reduction in gastrocnemius-to-body weight ratio at the study endpoint, whereas orlistat exhibited no obvious protective effects on relative muscle mass (Figure 6H). Together, these findings suggest that KBD3536 mitigates HFD-induced musculoskeletal dysfunction, effectively preserving relative muscle mass and exercise endurance.

### 2.7. KBD3536 Attenuates Early-Stage Glycemic Dysregulation in HFD-Fed Mice

**Figure 7:**
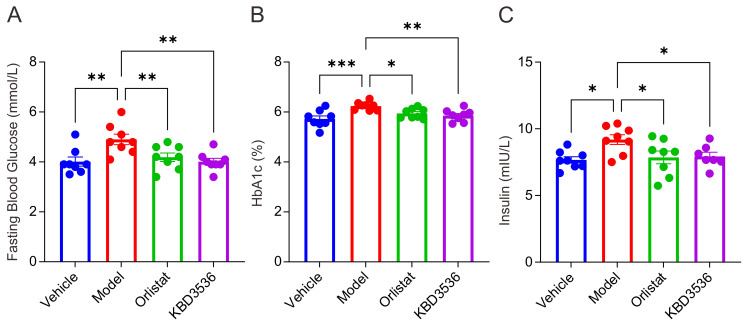
KBD3536 mitigates HFD-induced dysregulation of glycemic markers. (**A**) Fasting blood glucose (FBG) levels measured from tail vein blood after 7 weeks of treatment. (**B**) Whole-blood glycated hemoglobin (HbA1c) and (**C**) fasting serum insulin levels assessed at the experimental endpoint. Data are presented as mean ± SEM (*n* = 8 per group, except for the KBD3536 group in panel (**C**) where *n* = 7 due to technical failure during sample processing). Individual data points are shown as overlaid symbols. Statistical significance was determined by ordinary one-way ANOVA followed by Dunnett’s multiple comparisons test. * *p* < 0.05, ** *p* < 0.01, *** *p* < 0.001 vs. model group.

To evaluate the effects of KBD3536 on glycolipid metabolism, we assessed key glycemic indicators, including fasting blood glucose (FBG), whole-blood glycated hemoglobin (HbA1c), and fasting serum insulin. Compared with the HFD-fed model group, KBD3536 significantly reduced FBG at week 7 (Figure 7A), as well as endpoint HbA1c (Figure 7B) and fasting serum insulin levels (Figure 7C). These values approached those observed in the vehicle group. Because treatment was initiated during the early stages of obesity induction, the metabolic profile of the model group had not yet progressed to overt diabetes. Nevertheless, KBD3536 attenuated the mild but statistically significant HFD-induced elevations in glycemic markers, supporting a potential role in early metabolic intervention against prodromal insulin resistance.

We also assessed systemic lipid profiles, including serum total cholesterol (TC) and triglycerides (TG) (Figure S6). Although HFD feeding significantly increased circulating TC levels, neither KBD3536 nor orlistat significantly reversed this parameter. Notably, HFD feeding did not induce a significant elevation in serum TG levels at this stage; however, orlistat treatment led to a sharp increase in TG levels. In contrast, KBD3536-treated mice maintained baseline TG levels comparable to both the vehicle and model groups. Together, these findings suggest that KBD3536 improves early glycemic control without inducing the hypertriglyceridemia side effect observed with orlistat treatment.

### 2.8. KBD3536 Attenuates Hepatic Steatosis and Localized Inflammation

**Figure 8:**
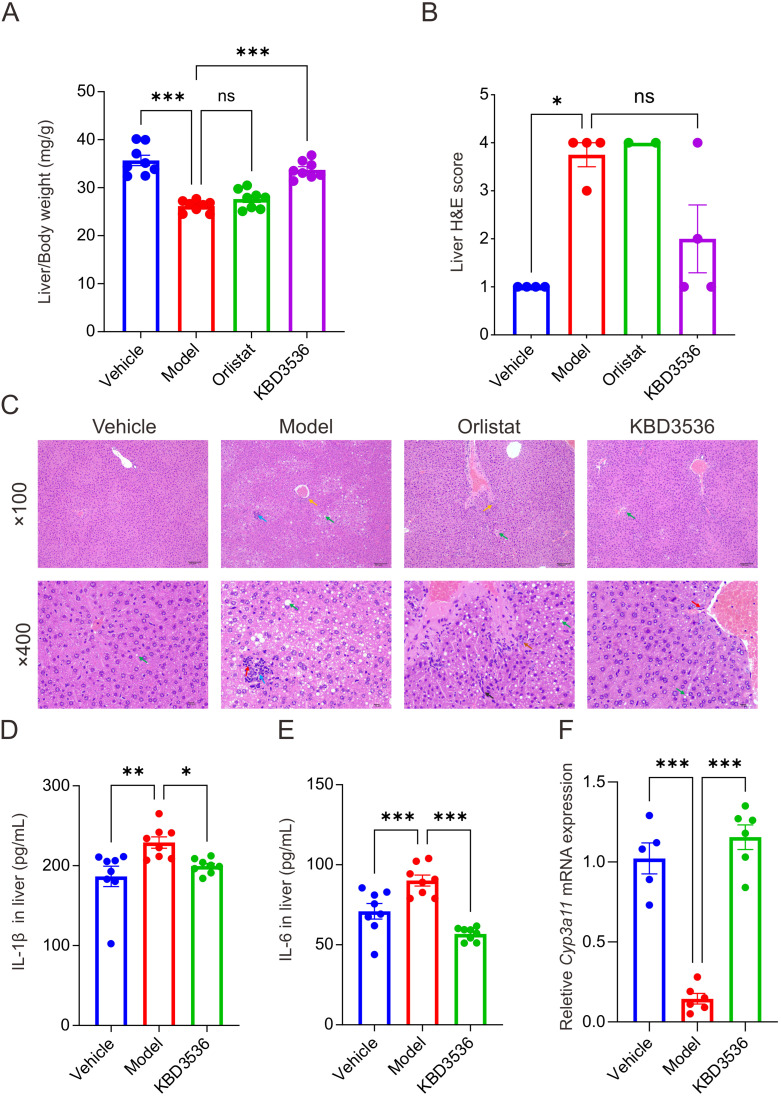
KBD3536 protects the liver against HFD-induced injury and inflammation. (**A**) Liver-to-body weight ratio at the experimental endpoint. (**B**) Histological assessment of hepatic steatosis, ballooning, and fibrosis based on the NAFLD activity score (NAS) system. (**C**) Representative H&E staining images of liver tissues (scale bars: 100 μm for ×100, and 10 μm for ×400). Green, blue, yellow, brown, black, and red arrows indicate hepatic steatosis, hepatocyte necrosis, fibrous tissue hyperplasia, fibroblasts, fibrocytes, and neutrophil infiltration, respectively. (**D**,**E**) Hepatic protein levels of (**D**) IL-1β and (**E**) IL-6 quantified by ELISA. (**F**) Relative *Cyp3a11* mRNA expression in liver tissues, determined by RT-qPCR and normalized to *Actb*. Data are presented as mean ± SEM (*n* = 4 per group for (**B**) (except for the Orlistat group where *n* = 2); *n* = 8 per group for (**A**,**D**,**E**); *n* = 6 per group for (**F**) (except for the KBD3536 group where *n* = 5 due to technical failure during sample processing)). Individual data points are shown as overlaid symbols. Statistical significance was analyzed using an ordinary one-way ANOVA followed by Dunnett’s multiple comparisons test for (**A**,**D**–**F**), and the Kruskal–Wallis test followed by Dunn’s multiple comparisons test for (**B**). * *p* < 0.05, ** *p* < 0.01, *** *p* < 0.001 vs. model group; ns, not significant.

The liver is a major metabolic organ vulnerable to HFD-induced lipotoxicity, commonly manifested as hepatic steatosis and localized inflammation [47,48]. To evaluate the effects of KBD3536 on obesity-induced hepatic injury, we performed a comprehensive assessment. Grossly, KBD3536 treatment partially restored the HFD-induced reduction in the liver-to-body weight ratio (Figure 8A). Histological analysis of H&E-stained sections revealed that the HFD-fed model group exhibited marked steatosis and inflammatory infiltration, consistent with early-stage metabolic dysfunction. Importantly, KBD3536 visibly attenuated these hepatic lipid accumulations and cellular infiltrations (Figure 8C), which was corroborated by a favorable downward trend in the semi-quantitative composite liver lesion score (Figure 8B). In contrast, orlistat exhibited minimal histological improvement in this context.

We next assessed the local inflammatory microenvironment within the liver. Because lipotoxicity-induced inflammasome activation promotes the release of pro-inflammatory cytokines, the hepatic levels of IL-1β and IL-6 were evaluated by ELISA. KBD3536 significantly reduced HFD-induced elevations in hepatic IL-1β (Figure 8D) and IL-6 (Figure 8E). Meanwhile, serum levels of liver injury biomarkers, alanine aminotransferase (ALT) and aspartate aminotransferase (AST), remained largely unaltered across all groups (Figure S7), suggesting that these localized inflammation represent an early-stage manifestation prior to overt, detectable hepatocellular necrosis.

To further evaluate specific aspects of hepatic metabolic capacity, we assessed *Cyp3a11*, the murine ortholog of human CYP3A4 and a cytochrome P450 enzyme involved in xenobiotic and lipid metabolism [49,50]. RT-qPCR analysis revealed that HFD feeding markedly suppressed *Cyp3a11* mRNA expression by approximately seven-fold; strikingly, KBD3536 treatment fully restored its expression to baseline levels (Figure 8F). Together, these findings suggest that KBD3536 alleviates early-stage HFD-induced hepatic inflammation and steatosis, while effectively rescuing the downregulation of key metabolic enzymes.

## 3. Discussion

Despite increasing interest in pharmacological NLRP3 inhibition, translating promising preclinical findings into sustained and clinically meaningful benefit remains challenging. In this study, we systematically evaluated the in vivo efficacy of KBD3536, a novel NLRP3 inhibitor, across a spectrum of sterile inflammatory conditions associated with distinct damage-associated molecular patterns (DAMPs) and NLRP3 activation. Rather than focusing on a single pathology, we strategically selected four interrelated in vivo models to evaluate the potential of KBD3536 across diverse pathological contexts: from acute crystalline-driven inflammation (MSU-induced air pouch and gouty arthritis models), to subacute physical tissue injury (radiation-induced dermatitis, RID), and finally to chronic metabolic stress (HFD-induced obesity). These preclinical findings highlight the broad applicability and translational promise of KBD3536 across diverse NLRP3-related pathologies and support its further translational investigation.

To establish an in vivo pharmacodynamic foundation for KBD3536, we first evaluated its activity in two independent MSU crystal-induced acute inflammation models, representing prototypical conditions driven by canonical DAMPs. Gouty inflammation represents a well-established model of crystalline-driven inflammation and has played a key role in defining the contribution of the NLRP3/IL-1β axis [9,15]. In the mouse air pouch model, oral administration of KBD3536 suppressed local IL-1β and IL-6 secretion, with effects comparable to those of MCC950. These local anti-inflammatory effects were further reflected in the rat gouty arthritis model, in which KBD3536 dose-dependently attenuated acute joint swelling and improved pain-related weight-bearing incapacitance. Together, these findings support the in vivo pharmacodynamic activity of KBD3536 and provide a rationale for its evaluation in more complex chronic and stress-induced inflammatory conditions.

Building upon the efficacy observed in acute crystalline inflammation, we next evaluated KBD3536 in a RID mouse model to explore its potential against physical stress-induced tissue injury. Radiotherapy-induced skin toxicity remains a major dose-limiting complication in oncology and is primarily managed with palliative topical corticosteroids, which often fail to halt the underlying pathogenic process [51,52]. Mechanistically, ionizing radiation promotes excessive reactive oxygen species (ROS) production, which can act as DAMPs to activate the NLRP3 inflammasome and contribute to dermal fibroblast activation and extracellular matrix remodeling [37,53]. Our in vivo findings with KBD3536 align with emerging evidence suggesting that pharmacological blockade of NLRP3 is a viable strategy to mitigate radiation-induced tissue damage [54,55]. In our study, systemic administration of KBD3536 attenuated radiation-induced inflammatory cell infiltration, accompanied by reductions in epidermal hyperplasia and structural dermal damage. Interestingly, we also observed a reduction in total NLRP3 protein levels in irradiated skin. This apparent downregulation may reflect disruption of the IL-1β autocrine/paracrine positive feedback loop previously described in inflammatory tissue remodeling [56,57]. Together, these findings support the potential of KBD3536 to mitigate radiation-induced skin injury.

Having demonstrated the efficacy of KBD3536 in physical stress-induced inflammation, we next investigated its early-intervention effects in high-fat diet (HFD)-induced obesity, where excess metabolic byproducts act as endogenous DAMPs. In this context, aberrant NLRP3 activation serves as a key driver of chronic metabolic inflammation. An important finding of this study is the apparent efficacy of KBD3536 under an early intervention regimen in restraining excessive adiposity and pathological weight gain. Preclinical studies in the anti-obesity field often evaluate therapeutic efficacy in established disease states, with treatment initiated after obesity and insulin resistance are already well developed [18,58,59,60,61]. Within the context of NLRP3-targeted therapeutics, recent evidence suggests that reversing chronic weight gain and adipose inflammation is challenging and may require either central nervous system (CNS)-penetrant inhibitors to modulate hypothalamic pathways or combination approaches involving glucagon-like peptide-1 (GLP-1) receptor agonists [19,20,21,62]. Against this background, our findings suggest that early pharmacological intervention with KBD3536, initiated at the onset of HFD exposure, may attenuate some aspects of lipotoxicity-associated inflammation and metabolic dysregulation. By limiting inflammatory signaling during the early stages of metabolic stress, KBD3536 appeared to restrict pathological adipose tissue expansion and was associated with mitigated deterioration in systemic metabolic parameters.

Apart from controlling excessive body weight gain, maintaining physical capacity and preserving lean body mass are vital components when evaluating metabolic interventions [24,63]. In this context, our findings suggest that KBD3536 exhibited a favorable profile by preferentially restricting adipose accumulation. Mice treated with KBD3536 maintained a higher gastrocnemius muscle-to-body weight ratio than HFD controls, accompanied by improvements in motor coordination, grip strength, and treadmill endurance. Importantly, these effects occurred without significant alterations in cumulative food intake, suggesting that the benefits of KBD3536 are unlikely to be driven primarily by appetite suppression. Mechanistically, unlike orlistat, which blocks intestinal lipid absorption [41], KBD3536 may act by favorably modulating the local inflammatory environment and partially restore AMPKα1 mRNA expression within adipose tissue, thereby contributing to the preservation of skeletal muscle function and overall motor performance during early dietary intervention.

Moreover, KBD3536 demonstrated the capacity to attenuate hepatic inflammation in HFD-fed mice. Metabolic dysfunction-associated steatohepatitis (MASH) is a common hepatic complication of prolonged dietary stress, characterized by persistent localized inflammation in which NLRP3 activation and the subsequent release of pro-inflammatory cytokines, particularly IL-1β and IL-6, play an important role [48,64]. In our study, KBD3536 treatment reduced hepatic steatosis and attenuated the overall hepatic inflammatory response, as evidenced by significantly lower local IL-1β and IL-6 levels. These observations are consistent with previous reports showing that pharmacological inhibition of NLRP3 attenuates liver inflammation and fibrosis in experimental models [65]. Beyond these changes, KBD3536 also modulated the expression of *Cyp3a11*. Chronic obesity and associated inflammatory stress are known to suppress hepatic drug-metabolizing enzymes, notably CYP3A4 (the human ortholog of murine CYP3A11), thereby impairing hepatic metabolic and detoxification functions [66,67]. Our findings suggest that KBD3536 partially restored the mRNA expression levels of *Cyp3a11* in HFD-fed mice, suggesting a potential role in support of CYP3A11-mediated metabolic pathways under dietary stress.

From a translational perspective, the broad activity of KBD3536 across multiple disease models highlights its potential relevance to various inflammasome-mediated pathologies. Its ability to suppress acute crystalline inflammation supports further evaluation in acute inflammatory settings, whereas the findings in radiation-induced tissue injury suggest potential utility in mitigating treatment-associated damage. Of particular interest, the early intervention strategy in the HFD model underscores the potential value of targeting NLRP3-mediated metabolic dysfunction before severe tissue remodeling occurs. For conditions reminiscent of early-stage metabolic syndrome, NLRP3 inhibition may represent a promising strategy to mitigate localized inflammatory and metabolic stress. Although these pathological conditions are etiologically distinct, they converge, at least in part, on aberrant NLRP3 activation which likely underlies the broad activity observed with KBD3536.

Despite these promising preclinical findings, several limitations of the present study warrant consideration. First, the in vivo administration protocol primarily reflects an early intervention strategy, necessitating future studies with delayed treatment regimens to fully evaluate its therapeutic efficacy in established advanced diseases. Second, while downstream inflammatory readouts support NLRP3 inhibition, direct in vivo target engagement and the exact decoupling of direct metabolic regulation versus secondary benefits from inflammation resolution require further investigation. Finally, as the current study primarily focused on proof-of-concept efficacy, the lack of systemic exposure data limits our ability to fully justify the dose selection. Consequently, evaluating broader multiple-dose regimens and fully characterizing the pharmacokinetic/pharmacodynamic (PK/PD) profiles will be a priority for next-stage investigations.

Nevertheless, the breadth of in vivo findings presented here supports the continued preclinical evaluation of this novel compound. In conclusion, KBD3536 demonstrated pharmacological activity across a diverse spectrum of NLRP3 -related models, supporting its further investigation as a promising candidate for NLRP3-related diseases. These findings highlight the translational promise of NLRP3 inhibition and provide a foundation for the future development of next-generation NLRP3 inhibitors.

## 4. Materials and Methods

### 4.1. Reagents and Antibodies

Compounds and Solvents: KBD3536 and MCC950 were provided by Kangbaida (Sichuan) Biotechnology Co., Ltd. (Chengdu, China). Orlistat (PHR1445), monosodium urate (MSU; U2875-5G), dimethyl sulfoxide (DMSO; D1435), and Solutol (102442756) were purchased from Sigma-Aldrich (St. Louis, MO, USA). Methylcellulose (M112866) was obtained from Aladdin (Shanghai, China). Kolliphor HS-15 (35907288Q0) was provided by BASF SE (Ludwigshafen, Germany) or Beijing Fengli Jingqiu Pharmaceutical Co., Ltd. (Beijing, China). Isoflurane was purchased from RWD Life Science (Shenzhen, China).

Detection Kits: Mouse IL-6 (88-7064-88) and IL-1β (88-7013A-88) uncoated ELISA kits were obtained from Invitrogen (Carlsbad, CA, USA). The insulin detection kit (ZC-38920) was purchased from Shanghai Zhuocai Biotechnology Co., Ltd. (Shanghai, China), and the glycated hemoglobin (HbA1c) kit (A056-1-1) was obtained from Nanjing Jiancheng Bioengineering Institute (Nanjing, China). The Absolutely RNA Miniprep Kit (5158-5999) was purchased from Agilent Technologies (Santa Clara, CA, USA). The HiScript^®^ III 1st Strand cDNA Synthesis Kit (R312-02) was obtained from Vazyme (Nanjing, China), and iTaq Universal SYBR Green Supermix (L001752) was purchased from Bio-Rad Laboratories (Hercules, CA, USA).

Antibodies: Primary antibodies used in this study included rabbit anti-NLRP3 polyclonal antibody (PA5-79740; Thermo Fisher Scientific, Waltham, MA, USA) and goat anti-mouse IL-1β polyclonal antibody (AF-401-NA; R&D Systems, Minneapolis, MN, USA). Secondary antibodies included Leica ImmunoDetector Reagent (DS9800; polymerized HRP anti-rabbit IgG; Leica Biosystems, Nussloch, Germany) and Vector HRP-labeled anti-goat IgG (MP-7405; Vector Laboratories, Newark, CA, USA).

### 4.2. Animals and Ethics Statement

SPF-grade male Sprague-Dawley rats (180–200 g, 6–7 weeks old) were purchased from the Experimental Animal Management Department of the Shanghai Institute of Family Planning Science (Shanghai, China). SPF-grade C57BL/6J mice (6–8 weeks old) were obtained from GemPharmatech Co., Ltd. (Chengdu, China) or Beijing Vital River Laboratory Animal Technology Co., Ltd. (Zhejiang, China).

All animal procedures were performed in strict accordance with the internationally accepted 3Rs (Replacement, Reduction, and Refinement) principles and the *Guide for the Care and Use of Laboratory Animals* (8th edition, National Academies Press). Mice were housed at Kangbaida (Sichuan) Biotechnology Co., Ltd.(Chengdu, China), and rats were maintained at PharmaLegacy Laboratories (Shanghai) Co., Ltd (Shanghai, China). Animals were housed in a controlled environment with an ambient temperature of 20–26 °C, relative humidity of 40–70%, and a pressure gradient of 11.4 Pa under a 12 h light/dark cycle, with ad libitum access to standard chow and water. The animal study protocols for the air pouch model, radiation-induced mouse dermatitis model, HFD-induced mouse obesity model, and short-term tolerability assessment in healthy lean mice were approved by the Institutional Animal Care and Use Committee (IACUC) of Kangbaida (Sichuan) Biotechnology Co., Ltd. (protocol codes: 20220602-6, 20240306-02, 20230713-06, and 20260414-04, respectively). The protocol for the MSU crystal-induced rat model of acute gouty arthritis was approved by the IACUC of PharmaLegacy Laboratories (Shanghai) Co., Ltd. (protocol code: PL22-0220-1).

### 4.3. Disease Models and Drug Administration

MSU-induced mouse air pouch model: Male C57BL/6J mice (8 weeks old) were shaved on the back, and dorsal air pouches were established by subcutaneous injection of 4 mL of sterile air on days 1 and 4. On day 4, the mice were assigned to three groups (*n* = 9/group) based on body weight: a model group, a KBD3536 group (50 mg/kg), and an MCC950 group (50 mg/kg), with an additional naïve group included. From day 5 to day 7, treatments were administered via oral gavage twice daily (BID). The vehicle, consisting of 10% DMSO, 10% Solutol, and 80% purified water (*v*/*v*/*v*), was administered to both the model and naïve groups. On day 7, 0.5 h after the morning administration, an MSU suspension (1 mL, 3 mg/mL) was injected into the air pouch, and the experiment was terminated 5 h post-injection.

MSU crystal-induced rat model of acute gouty arthritis: Male SD rats weighing 180–200 g were randomized into a normal control group, an MSU model group, a positive control group (MCC950, 50 mg/kg), and high- or low-dose KBD3536 groups (50 or 15 mg/kg) (*n* = 12 per group). Starting from day 1, drugs were administered via oral gavage once daily (QD) for 9 consecutive days. The vehicle used was DMSO: HS-15: purified water (5:10:85, *v*/*v*/*v*), and the administration volume was 10 mL/kg. On day 7, 30 min after drug administration, acute gouty arthritis was induced in all groups except the normal control group by a single intra-articular injection of 50 μL of MSU suspension (120 mg/mL) into the right hind knee joint.

Radiation-induced mouse dermatitis model: Female C57BL/6J mice (6 weeks old) were randomly divided into a control group, a model group, and a KBD3536 group (*n* = 10/group) based on body weight. On day 0, mice in the model and treatment groups received a single localized X-ray irradiation (20 Gy, 160 KV, 25 mA) to induce acute radiation dermatitis. Starting from day 1, mice in the KBD3536 group were administered KBD3536 (100 mg/kg) via oral gavage once daily (QD) for 35 consecutive days. The administration volume was 10 mL/kg. The control and model groups were given an equivalent volume of the vehicle (DMSO: HS-15: purified water = 5:10:85, *v*/*v*/*v*).

HFD-induced obesity mouse model: Male C57BL/6J mice (6 weeks old) were allocated to a normal control group (fed a standard chow diet) and a model group (fed a 60% kcal high-fat diet [HFD]). Once a statistically significant difference in body weight emerged (on day 10), the HFD-fed mice were randomized into a model group (HFD + vehicle), a positive control group (HFD + orlistat), and a KBD3536 group (HFD + KBD3536), with 8 mice per group. The KBD3536 group received oral administration of the compound at 100 mg/kg twice daily (BID). The orlistat group received oral administration once daily (QD) at 20 mg/kg for the first two weeks, which was subsequently adjusted to 50 mg/kg. Treatments continued for 58 days with an administration volume of 5 mL/kg. The normal control and model groups received an equal volume of vehicle (DMSO: HS-15: 0.5% MC = 5:10:85, *v*/*v*/*v*).

Short-term tolerability assessment in healthy lean mice: Male C57BL/6J mice (7 weeks old) were randomly assigned to a control group and a KBD3536 treatment group. The KBD3536 group received oral administration of the compound at 100 mg/kg twice daily (BID) for 14 consecutive days, while the control group received an equal volume of vehicle (DMSO: HS-15: 0.5% MC = 5:10:85, *v*/*v*/*v*).

### 4.4. In Vivo Efficacy and Behavioral Assessments

Assessment of arthritis symptoms: During the experimental period, rat body weights were recorded on days 1, 4, and 9. At 4, 24, and 48 h after intra-articular injection of MSU or saline, a systematic evaluation of joint symptoms was performed as follows:

Gait Pain Assessment: Pain-related functional impairment was evaluated using an incapacitance tester (IITC Life Science, Woodland Hills, CA, USA; Cat# 600MR) to measure the difference in weight-bearing distribution between the hind paws. For each measurement, the rat was carefully placed in the testing chamber, and the static weight (in grams) borne by each hind paw was independently recorded. The weight-bearing deficit was calculated as the difference in force exerted by the uninjured left paw and the MSU-injected right paw (Left minus Right). Each rat was assessed five independent times, and the average difference was calculated to represent the degree of joint pain and incapacitance.

Surface Temperature Measurement: The surface temperature of the right hind knee joint was recorded using a standard thermometer.

Joint Swelling Measurement: Rats were anesthetized with 1–4% inhaled isoflurane for 3–5 min. The transverse diameter (medial-to-lateral distance) of the right hind knee joint was measured using a vernier caliper to assess the degree of joint swelling.

Skin lesion scoring: During the administration period in the radiation-induced mouse dermatitis model, body weight was recorded twice weekly, and the rate of body weight change was calculated. The severity of skin lesions at the irradiated site was evaluated using a blinded, semi-quantitative clinical scoring system ranging from 0 to 4. The scoring criteria were defined as follows:

• 0: Normal skin.
• 0.5: Small area of scaling (appearance of flaking).
• 1: Larger area of scaling (covering <1/4 of the chest area).
• 1.5: Extensive scaling (covering >1/4 of the chest area).
• 2: Small area of mild desquamation (smooth epidermis) or mild crusting (thickened, rough skin).
• 2.5: Larger area of mild desquamation or crusting (covering >1/2 of the affected area).
• 3: Small area of severe desquamation (depressed epidermis, scab-like appearance, nearly complete loss of the epidermal layer).
• 3.5: Larger area of severe desquamation (covering >1/4 of the affected area).
• 4: Extensive severe desquamation (covering the majority of the chest and back), accompanied by moist exudation and erythema.

Assessment of obesity-related physiological parameters and motor performance: During the treatment period, body weight and food intake were recorded twice per week, and the rate of body weight change and energy intake were calculated. Following the final administration, mice were fasted overnight, after which fasting body weight was recorded. Body length (measured from the tip of the nose to the base of the tail) was determined under anesthesia to calculate Lee’s index using the following formula:

(1) $$Lee^{\prime }s\;index=\frac{\sqrt[{3}]{body\;weight\left(g\right)}\times 1000}{body\;length(cm)}$$

Body weight change rate (%) was calculated using the following formula:

(2) $$Body\;weight\;change\;rate\left(\%\right)=\frac{W_{t}-W_{0}}{W_{t}}\times 100\%$$

where *W*_t_ is the body weight at a specific evaluation time point, and *W*_0_ is the initial body weight on Day 0.

Relative change in weight gain (%) was calculated using the following formula:

(3) $$Relative\;change\;in\;weight\;gain\left(\%\right)=\frac{RT-RM}{RM}\;\times 100$$

where *R*_T_ is treatment group weight change rate (%) at endpoint, and *R*_M_ is Model group weight change rate (%) at endpoint.

Grip Strength Test: Forelimb grip strength was assessed at week 6 of treatment using a grip strength meter (KW-ZL-1; Nanjing Calvin Biotechnology Co., Ltd., Nanjing, China). Each animal underwent 3 independent trials, and the average grip strength and maximum grip strength-to-body weight ratio were calculated.

Rotarod Test: Motor coordination was evaluated at week 7 using a rotarod apparatus (ZH-600B; Anhui Zhenghua Biological Instrument Equipment Co., Ltd., Anhui, China). Mice underwent acclimation training for two consecutive days prior to the formal test. The formal test was conducted at an initial speed of 5 rpm, accelerating to 40 rpm over 5 min, with a total test duration of 10 min. Latency to fall was recorded for each mouse.

Treadmill Exhaustion Test: Endurance was assessed at week 8 using a treadmill system (XR-PT-10B; Shanghai Xinsoft Technology Co., Ltd., Shanghai, China). Mice underwent habituation training for three consecutive days prior to the formal exhaustion test. The test protocol involved an initial speed of 10 m/min, followed by acceleration and maintenance for 5 min within a 3 min window, followed by acceleration to 18 m/min over 15 min with maintenance for an additional 15 min. To encourage continuous running, mild electrical stimulation (0.4 mA, 10 s per pulse, up to 50 times) was applied to the tail. Time to exhaustion, total distance traveled, and exhaustion speed were recorded.

### 4.5. Sample Collection

At the end of the experiments, all animals were euthanized (mice via CO_2_ inhalation and rats via CO_2_ inhalation followed by cervical dislocation). Rats in the MSU-induced acute gouty arthritis model were euthanized immediately after completion of the behavioral assessments, and no tissue samples were collected. Sample collection for the other models was performed as follows:

MSU-induced mouse air pouch model: The air pouches were lavaged by injection of 1 mL of PBS. The resulting air pouch exudates were collected and stored at −80 °C for subsequent ELISA analysis.

HFD-induced obesity mouse model: Blood was collected from the tail tip to measure blood glucose levels at week 7. At the experimental endpoint, whole blood was collected for serum and plasma separation and stored at −80 °C. The liver, epididymal fat, perirenal fat, and gastrocnemius muscle were harvested and weighed. Portions of these tissues were fixed in 10% neutral buffered formalin for histopathological examination, while the remaining tissues were snap-frozen in liquid nitrogen and stored at −80 °C for RNA extraction and ELISA testing.

Radiation-induced mouse dermatitis model: Skin tissues from the irradiated site were collected. A portion of the tissue was fixed in 10% formalin for pathological analysis, while the remainder was frozen for RNA extraction and ELISA testing.

### 4.6. Hematological and Serum Biochemical Analysis

Fasting blood glucose levels were monitored using a OneTouch blood glucose meter (LifeScan, Inc., Milpitas, CA, USA). Serum levels of triglycerides (TG), total cholesterol (TC), aspartate aminotransferase (AST), and alanine aminotransferase (ALT) were determined using a fully automated veterinary biochemistry analyzer (BS-240VET; Mindray, Shenzhen, China). Insulin and glycated hemoglobin (HbA1c) levels were measured using commercial assay kits according to the manufacturers’ instructions. Routine blood parameters were analyzed using a five-part differential veterinary hematology analyzer (DF 55Vet; Dymind, Shenzhen, China).

### 4.7. Histopathology and Immunohistochemistry

Pathological and immunohistochemical analysis of skin tissues from the radiation-induced mouse dermatitis model:

Tissue Processing and Automated Sectioning: Skin tissues from the irradiated sites were rinsed under running water for 4 h, dehydrated using an automated tissue processor (VP1-JC; Sakura Finetek, Torrance, CA, USA), and embedded in paraffin using an embedding station (TEC5 CM JC-2; Sakura). Serial sections with a thickness of 4 μm were prepared using a rotary microtome (HistoCore Multicut; Leica Biosystems, Nussloch, Germany). All subsequent staining procedures for skin sections were conducted using automated staining platforms.

Automated H&E and Masson’s Trichrome Staining: Sections were deparaffinized in xylene and rehydrated through a graded ethanol series prior to automated staining. For H&E staining, sections were incubated with hematoxylin for 7 min, followed by differentiation and bluing, and then counterstained with eosin for 30 s. For Masson’s trichrome staining to evaluate skin fibrosis, sections were sequentially stained with Weigert’s iron hematoxylin (5–10 min), Biebrich scarlet-acid fuchsin (5–10 min), and aniline blue (3–5 min), followed by differentiation in 1% acetic acid for 1 min. After staining, sections underwent routine dehydration, clearing, and mounting.

Automated Immunohistochemistry (IHC) Staining: NLRP3 and IL-1β expression in skin sections was evaluated using a Leica Bond automated IHC stainer. Heat-induced antigen retrieval was performed using Bond ER1/ER2 buffer at 100 °C for 20 min, and endogenous peroxidase activity was blocked with 3% hydrogen peroxide for 10 min. Subsequently, sections were incubated with rabbit anti-NLRP3 (1:250) or goat anti-IL-1β (1:40) polyclonal primary antibodies at room temperature for 60 min. After washing, sections were incubated with an HRP-labeled goat anti-rabbit secondary antibody at room temperature for 8 min. Signal development was performed using DAB for 10 min, followed by counterstaining with hematoxylin for 5 min, dehydration, and mounting.

Image Scanning and Quantitative Evaluation: All H&E-, Masson’s trichrome-, and IHC-stained sections were digitally scanned using a Pannoramic SCAN slide scanner (3DHISTECH Ltd., Budapest, Hungary). Image analysis was performed using the HALO™ software platform (version 3.4.2986.254; Indica Labs, Albuquerque, NM, USA).

Inflammation and Pathological Scoring: Epidermal thickness was measured using HALO™ software. The degree of inflammatory tissue damage in H&E sections was evaluated using a blinded 0–5 scoring system (Grade 0 = no inflammatory response; Grade 5 = extremely severe inflammatory response).

Fibrosis Assessment: Skin fibrosis in Masson’s trichrome-stained sections was evaluated using the modified Ashcroft scoring criteria.

IHC Quantitative Analysis: The number of NLRP3- or IL-1β-positive cells and the total tissue area were automatically identified and quantified. IHC results were expressed as positive cell density (n/mm^2^), calculated as follows: positive cell density = number of positive cells/tissue area (mm^2^).

Morphological and pathological analysis of tissues from the high-fat diet (HFD)-induced mouse obesity model (liver and adipose tissue):

Tissue Processing and H&E Staining: Liver and adipose tissues fixed in neutral buffered formalin were processed using an automated tissue processor through a graded ethanol series for dehydration and xylene for clearing, followed by routine paraffin embedding and sectioning. Sections were deparaffinized, rehydrated, and stained with hematoxylin and eosin (H&E). Briefly, sections were stained with hematoxylin for 5–10 min, differentiated in acid-alcohol for approximately 3 s, and blued in a weak alkaline aqueous solution. Subsequently, sections were counterstained with alcohol-soluble eosin for 3 min. Finally, slides were dehydrated through graded ethanol, cleared in xylene, and mounted with neutral resin.

Image Acquisition and Data Analysis: Stained sections were observed using a digital trinocular camera microscope (BA210Digital; Motic Industrial Group Co., Ltd., Xiamen, China). Image acquisition and measurements were performed using Motic Images Advanced 3.2 software. The area of adipocytes in adipose tissue were quantitatively analyzed. For liver tissues, sections were evaluated in a blinded manner by an experienced pathologist. The NAFLD activity score (NAS) and fibrosis staging, based on the scoring system established by Kleiner et al. [68], were used to assess the severity of hepatic steatosis, lobular inflammation, and hepatocyte ballooning, as well as the degree of liver fibrosis, respectively.

### 4.8. Enzyme-Linked Immunosorbent Assay (ELISA)

Liver and skin tissue samples harvested at the experimental endpoint were homogenized in pre-cooled PBS at a weight-to-volume ratio of 1:9. The homogenates were centrifuged at 12,000× *g* for 15 min at 4 °C to collect supernatants. Mouse air pouch exudate samples were thawed on ice and centrifuged to obtain supernatants for analysis. Subsequently, the concentrations of IL-6 (in air pouch exudates, liver tissues, and skin tissues) and IL-1β (in air pouch exudates and liver tissues) were measured using sandwich ELISA according to the manufacturers’ instructions. Absorbance was measured at 450 nm (reference wavelength: 570 nm) using a Varioskan LUX multimode microplate reader (Thermo Fisher Scientific, Waltham, MA, USA).

### 4.9. Quantitative Real-Time PCR (RT-qPCR)

RNA Extraction and cDNA Synthesis: Total RNA was extracted from liver and epididymal adipose tissues using the Absolutely RNA Miniprep Kit (Agilent Technologies, Santa Clara, CA, USA). RNA concentration and purity were assessed using a NanoPhotometer^®^ (N60; IMPLEN), ensuring an A260/A280 ratio of 1.8–2.1 and an A260/A230 ratio > 2.0. Subsequently, 1 μg of total RNA was reverse-transcribed into cDNA using the HiScript^®^ III 1st Strand cDNA Synthesis Kit (Vazyme, Nanjing, China). Reverse transcription was performed at 37 °C for 15 min, followed by heating at 85 °C for 5 s to inactivate reverse transcriptase.

RT-qPCR Amplification: RT-qPCR was performed on a CFX96 Real-Time PCR Detection System (Bio-Rad Laboratories, Hercules, CA, USA) using iTaq Universal SYBR Green Supermix (Bio-Rad). The 20 μL reaction mixture consisted of 1 μL of cDNA template, 10 μL of 2× SYBR Green Supermix, 200 nM of each forward and reverse primer, and nuclease-free water. Thermal cycling conditions included an initial denaturation at 95 °C for 3 min, followed by 40 cycles of denaturation at 95 °C for 10 s and annealing/extension at 60 °C for 30 s.

Data Collection and Analysis: All samples were run in technical duplicates. Melting curve analysis was performed post-amplification to verify primer specificity and rule out primer-dimer formation. Relative mRNA expression levels of target genes were calculated using the 2^−ΔΔCt^ method, with β-actin serving as the endogenous reference gene. Data acquisition and initial analysis were conducted using CFX Maestro software (version 2.0; Bio-Rad Laboratories, Hercules, CA, USA). Primer sequences used in this study are listed in Table 1.

### 4.10. Statistical Analysis

All statistical analyses were performed, and graphs were generated using GraphPad Prism software (version 10.6, GraphPad Software, San Diego, CA, USA). For continuous data, comparisons among multiple groups were analyzed using original one-way ANOVA followed by Dunnett’s multiple comparisons test, or Repeated Measures (RM) Two-way ANOVA followed by Dunnett’s multiple comparisons test for time-course data. For ordinal data, the non-parametric Kruskal–Wallis test followed by Dunn’s post hoc test was employed for multiple-group comparisons, while the Mann–Whitney U test was used for two-group comparisons. Due to the small sample size (*n* = 2), statistical analysis was omitted for the Orlistat group in the liver H&E staining assay. Data are primarily presented as the mean ± standard error of the mean (SEM). A *p*-value of < 0.05 was considered statistically significant. Levels of significance are denoted as * *p* < 0.05, ** *p* < 0.01, and *** *p* < 0.001.

## 5. Conclusions

In conclusion, this study demonstrates that the novel NLRP3 inhibitor KBD3536 exhibits broad anti-inflammatory and tissue-protective activity across diverse rodent models of acute crystalline arthritis, radiation dermatitis, and diet-induced obesity. By attenuating inflammation and ameliorating associated disease phenotypes in these distinct pathological settings, KBD3536 represents a promising candidate for further investigation in NLRP3-related diseases.

## Figures and Tables

**Table 1:** Primer Sequences.

Gene	Forward	Reverse
*Actb*	CCTAGGCACCAGGGTGTGA	GGTTGGCCTTAGGGTTCAGG
*Prkaa1*	ACCTGAGAACGTCCTGCTTG	GGCCTGCGTACAATCTTCCT
*Cyp3a11*	AACCTGGGTGCTCCTAGCAA	TTGACCATCAAACAACCCCC

## Data Availability

The original contributions presented in this study are included in the article/Supplementary Materials. Further inquiries can be directed to the corresponding authors.

## Supplementary Materials

The following supporting information can be downloaded at: https://www.mdpi.com/article/10.3390/ph19071083/s1, Figure S1: Body weight in the MSU crystal-induced rat model of acute gouty arthritis; Figure S2. Area under the curve (AUC) analysis of skin injury scores; Figure S3: Representative immunohistochemistry (IHC) images of NLRP3 and IL-1β in irradiated skin tissues; Figure S4: Dynamic assessment of food intake in the HFD-induced mouse model; Figure S5: Short-term tolerability assessment of KBD3536: No adverse impacts on physiological growth or food intake in healthy lean mice; Figure S6: Evaluation of circulating lipid levels in the HFD-induced obesity mouse model; Figure S7: Assessment of serum biomarkers for hepatic injury in the HFD-induced obesity mouse model.

## Abbreviations

The following abbreviations are used in this manuscript:

ALT	Alanine aminotransferase
ASC	Apoptosis-associated speck-like protein containing a CARD
AST	Aspartate aminotransferase
BID	Twice daily (Bis in die)
CAPS	Cryopyrin-associated periodic syndromes
CNS	Central nervous system
DAMPs	Danger-associated molecular patterns
DIO	Diet-induced obesity
DMSO	Dimethyl sulfoxide
ELISA	Enzyme-linked immunosorbent assay
eWAT	Epididymal white adipose tissue
FBG	Fasting blood glucose
GLP-1	Glucagon-like peptide-1
H&E	Hematoxylin and eosin
HbA1c	Glycated hemoglobin
HFD	High-fat diet
IHC	Immunohistochemistry
IL-1β	Interleukin-1β
IL-6	Interleukin-6
IL-18	Interleukin-18
Lym	Lymphocytes
MASH	Metabolic dysfunction-associated steatohepatitis
MC	Methylcellulose
Mon	Monocytes
MSU	Monosodium urate
NAFLD	Nonalcoholic fatty liver disease
NAS	NAFLD Activity Score
Neu	Neutrophils
NLRP3	NOD-like receptor family pyrin domain-containing 3
PBMCs	Peripheral blood mononuclear cells
pWAT	Perirenal white adipose tissue
QD	Once daily (Quaque die)
RID	Radiation-induced dermatitis
ROS	Reactive oxygen species
RT-qPCR	Quantitative real-time polymerase chain reaction
SEM	Standard error of the mean
TC	Total cholesterol
TG	Triglycerides
WAT	White adipose tissue
