# Supplementary material for: NLRP3 Inhibitor KBD3536 Attenuates Acute Inflammation, Radiation-Induced Skin Injury, and Early Metabolic Dysfunction in Preclinical Models
Source: Pharmaceuticals (Basel). 2026 Jul 14;19(7):1083. doi: 10.3390/ph19071083 (PMC13414654; doi:10.3390/ph19071083)

Figure S1. Body weight in the MSU crystal-induced rat model of acute gouty arthritis. Body weights were monitored and recorded on Days 1, 4, and 9 of the experimental period. Data are expressed as mean ± SEM (n = 12 per group).

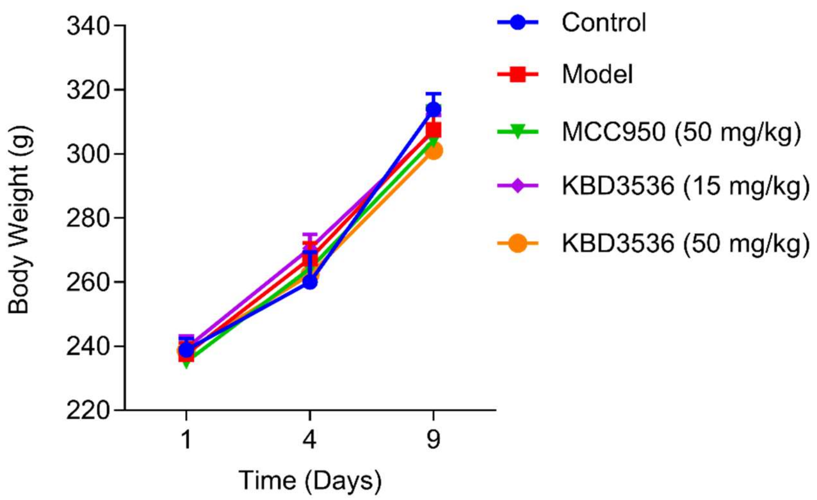

Figure S2. Area under the curve (AUC) analysis of skin injury scores. The bar graph represents the cumulative severity of skin injury over the monitoring period. Data are expressed as mean ± SEM (n = 10 per group). Statistical significance between the model and KBD3536-treated groups was determined using the non-parametric Mann-Whitney U test. $^{**}p < 0.01$ vs. model group.

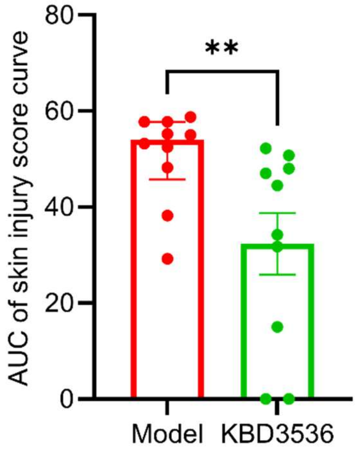

Figure S3. Representative immunohistochemistry (IHC) images of NLRP3 and IL-1β in irradiated skin tissues. Skin tissue sections were collected at the experimental endpoint. Tissues were subjected to IHC staining to visualize the local expression of NLRP3 and IL-1β. Scale bar = 100 μm.

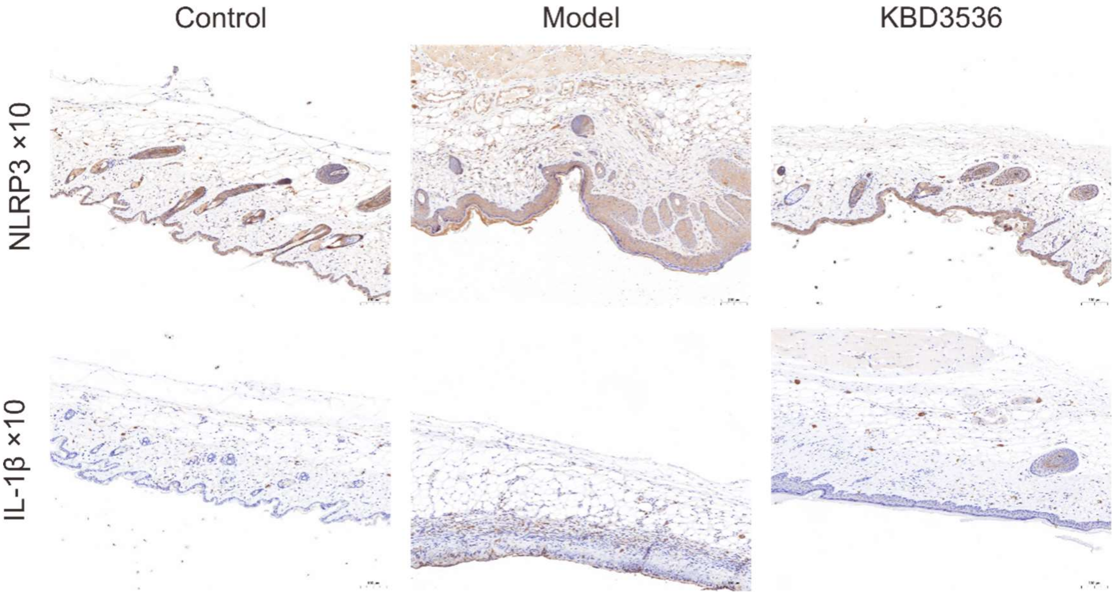

Figure S4. Dynamic assessment of food intake in the HFD-induced mouse model. Average daily food consumption (g/day/mouse) was recorded at the indicated time intervals throughout the 8-week (58-day) experimental period. Data are presented as mean ± SEM (n = 8 per group).

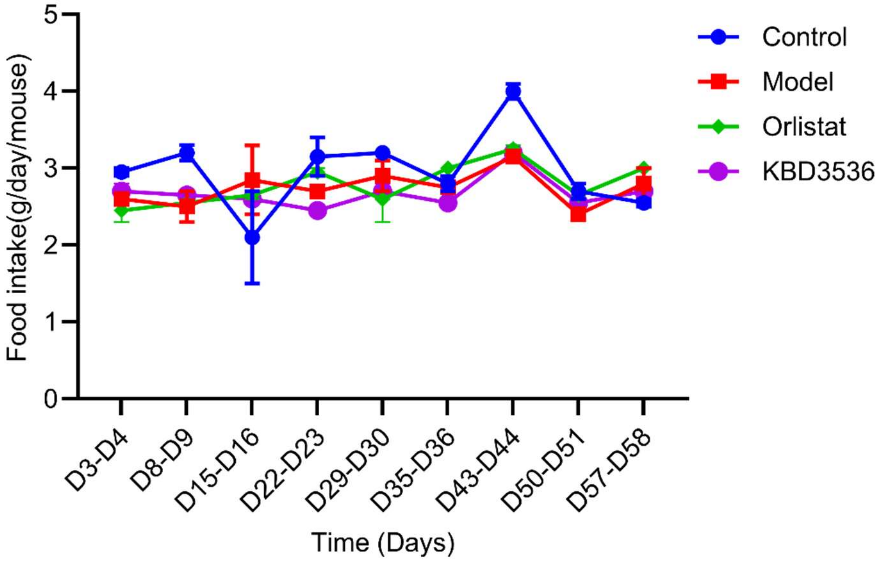

Figure S5. Short-term tolerability assessment of KBD3536: No adverse impacts on body weight or food intake in healthy lean mice. Normal, lean C57BL/6J mice were orally administered vehicle (DMSO: HS-15: 0.5% MC = 5:10:85, v/v/v) or KBD3536 (100 mg/kg) twice daily (BID) for a 14-day short-term regimen. **(A)** Absolute body weight (g) trajectories and **(B)** average daily food intake (g/day/mouse) were monitored at the indicated time points throughout the experimental period. Data are presented as mean ± SEM (n = 6 per group).

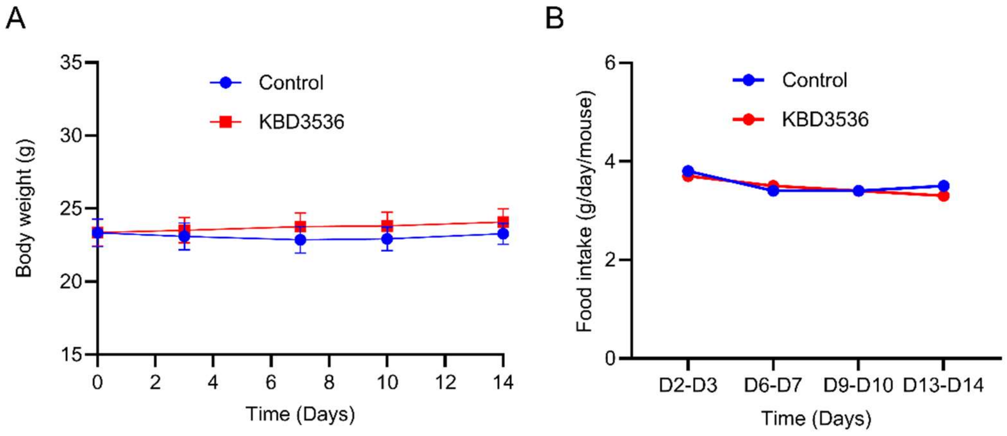

Figure S6. Evaluation of circulating lipid levels in the HFD-induced obesity mouse model. Serum levels of **(A)** total cholesterol (TC) and **(B)** triglycerides (TG) were assessed at the experimental endpoint. Data are presented as mean ± SEM (n = 8 per group). Statistical significance was determined by one-way ANOVA followed by Dunnett's multiple comparisons test. ***p < 0.001 vs. model group; ns, not significant.

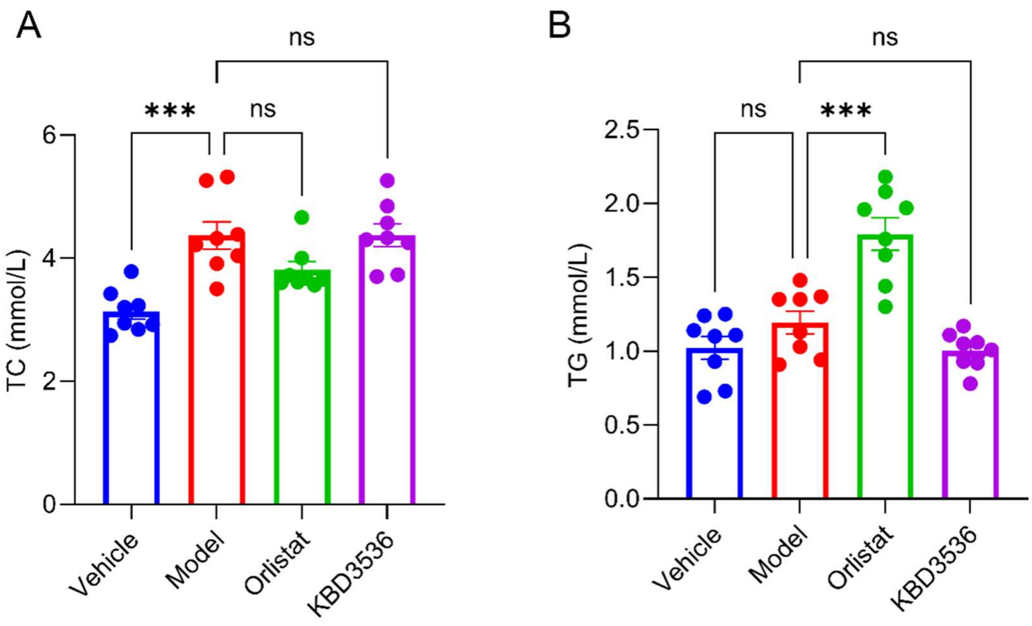

Figure S7. Assessment of serum biomarkers for hepatic injury in the HFD-induced obesity mouse model. Serum levels of **(A)** alanine aminotransferase (ALT) and **(B)** aspartate aminotransferase (AST) were evaluated at the experimental endpoint. Data are presented as mean ± SEM (n = 8 per group). Statistical significance was determined by one-way ANOVA followed by Dunnett's multiple comparisons test. ns, not significant.

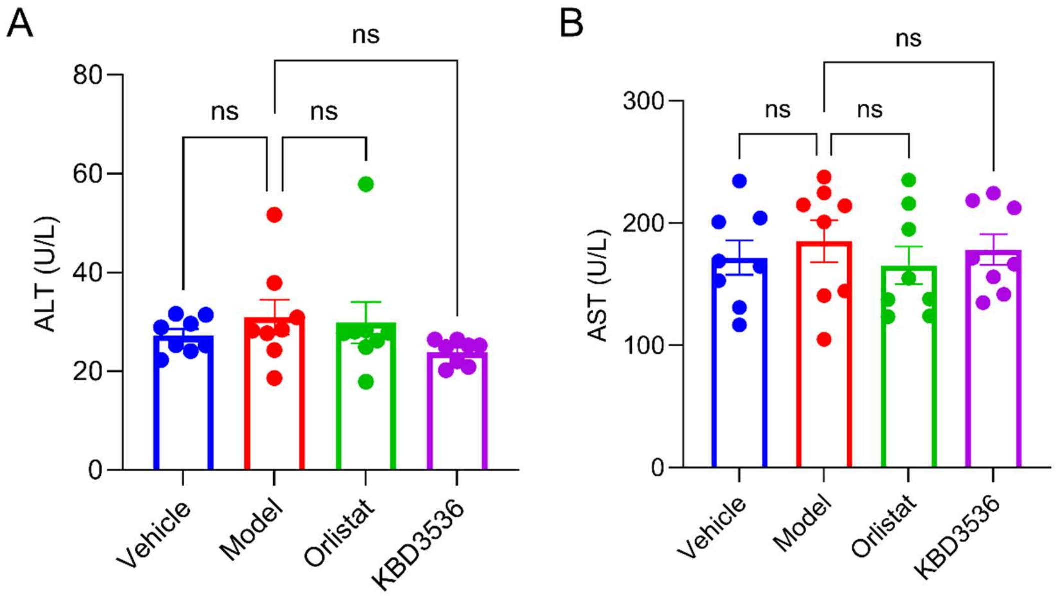

Supplement: Supplementary file 1 [file pharmaceuticals-19-01083-s001.zip › pharmaceuticals-4374167-supplementary.pdf]
